# Sequential stimulation with different concentrations of BMP4 promotes the differentiation of human embryonic stem cells into dental epithelium with potential for tooth formation

**DOI:** 10.1186/s13287-019-1378-7

**Published:** 2019-08-29

**Authors:** Qian Li, Siqi Zhang, Yi Sui, Xiaoming Fu, Yan Li, Shicheng Wei

**Affiliations:** 1 Department of Oral and Maxillofacial Surgery/Central Laboratory, Peking University School and Hospital of Stomatology, Beijing, 100081 China; 2 Laboratory of Biomaterials and Regenerative Medicine, Academy for Advanced Interdisciplinary Studies, Peking University, Beijing, 100871 China

**Keywords:** Human embryonic stem (hES) cells, Dental epithelium (DE), Bone morphogenetic protein 4(BMP4), Sequential, Tooth regeneration

## Abstract

**Background:**

Tooth loss caused by caries or injuries has a negative effect on human health; thus, it is important to develop a reliable method of tooth regeneration. Research on tooth regeneration has mainly focused on mouse pluripotent stem cells, mouse embryonic stem cells, and adult stem cells from various sources in mice, whereas little has examined the differentiation of human embryonic stem (hES) cells into dental epithelium (DE) and odontogenic potential in vivo.

**Methods:**

In this study, we induced hES cells to differentiate into dental epithelium using different concentrations of bone morphogenetic protein 4 (BMP4). With 1 pM BMP4, the hES cells differentiated into oral ectoderm (OE). These cells were then stimulated with 30 pM BMP4. Quantitative reverse transcription-polymerase chain reaction (qRT-PCR) and immunofluorescence showed the differentiation of OE and DE. The DE generated was mixed with embryonic day 14.5 mouse dental mesenchyme (DM) and transplanted into the renal capsules of nude mice. Thirty days later, the resulting tooth-like structures were examined by micro-computed tomography and hematoxylin and eosin staining.

**Results:**

After 4 days of 1 pM BMP4 stimulation, Pitx1-positive OE formed. qRT-PCR and immunofluorescence revealed that induction with 30 pM BMP4 for 2 days caused the OE to differentiate into Pitx2/Dlx2/AMBN-positive DE-like cells. These cells also expressed β-catenin and p-Smad1/5/8, which are key proteins in the Wnt/β-catenin and Bmp signaling pathways, respectively. Thirty days after in vivo transplantation, six teeth with enamel and dentin had formed on the kidney.

**Conclusions:**

These results show that hES cells differentiated into DE after sequential stimulation with different concentrations of BMP4, and the DE thus generated showed odontogenic potential.

**Electronic supplementary material:**

The online version of this article (10.1186/s13287-019-1378-7) contains supplementary material, which is available to authorized users.

## Background

Teeth are very important organs. Tooth loss affects not only facial esthetics but also chewing and pronunciation. Some oral diseases are also closely related to systemic diseases, such as diabetes and heart disease [1, 2]. Clinically, dentures, including implantable dentures, have been used to replace lost teeth, but they cannot always achieve the esthetic and functional effects of natural teeth [3, 4]. Therefore, the use of stem cell technology to produce regenerated teeth with the same morphology and function as natural teeth is the ideal way to solve the problem of tooth loss [5, 6].

In recent years, tooth regeneration has achieved gratifying results with the promotion of stem cells and regenerative medicine. The research on tooth regeneration has examined two main areas. Studies focusing on tooth morphology have used biological scaffolds as a skeleton and combined this with stem cells to obtain teeth with predetermined shapes [7]. Alternatively, based on the theory of embryonic tooth development, regenerative teeth can be obtained by inducing stem cells to simulate the natural process of tooth development. Tissue development and morphogenesis are complex processes. Tooth ontogenesis requires the exchange of information between the tooth germ epithelium and mesenchyme, and more than 300 genes are involved in this process [8]. Many members of the bone morphogenetic protein (BMP) family are involved in the development of tooth germ, including early morphogenesis, cell proliferation and differentiation, and germination [9]. BMP4 plays an important role in the formation and development of tooth germ. Initially, BMP4 is highly expressed in the epithelium and then in the mesenchyme, and the transfer of expression is accompanied by the transfer of odontogenic potential [10]. As a result, we postulate that BMP4 is associated with odontogenic potential and is a key factor in the mutual induction of epithelial and mesenchyme.

There is interplay between epithelium and mesenchyme in tooth development. Takashi Tsuji et al. developed a three-dimensional organ–germ culture method by which a bioengineered tooth was generated by reconstituting cells from the epithelial and mesenchymal tissues in embryonic day 14.5 (ED14.5) mice [11]. With this method, successful tooth restoration was realized in a postnatal canine model [12]. Yelick et al. fabricated three-dimensional (3D) dental epithelium (DE)–dental mesenchyme (DM) cell constructs of predetermined shape and size using human dental pulp cells and porcine DE cells with collagen gel and Matrigel [13]. They constructed full-size bioengineered 3D tooth buds with dental tissues and cells from postnatal porcine teeth and human wisdom tooth dental pulp, and the tooth buds ultimately formed dentin, enamel, and complete tooth crowns [14]. This indicates that epithelium in full contact with mesenchyme benefits odontogenesis.

The odontogenic potential of tooth germ starts in the epithelium, and ED10.5 tooth germ epithelium is the dental tissue with the greatest odontogenic potential [5]. However, there are some difficulties using ED10.5 tooth germ epithelium, including the small volume, operating difficulties, and limited cell source. More importantly, early tooth germ epithelium cannot be obtained from humans [15, 16]. As a result, scientists are trying to induce stem cells to form dental epithelial-like cells and hope to realize tooth regeneration. Makiko Arakak, a Japanese scientist, used the cervical-loop cells of rat tooth germ to establish ameloblast cell lines, which are dental epithelial stem cells. He co-cultured these cells with induced pluripotent stem (iPS) cells to obtain ameloblastoids that expressed P63, CK14, enamelin, and amelogenin [17]. Adult stem cells that can be used for tooth epithelial cell regeneration are mainly epidermal stem cells isolated from the basal layer of the skin and hair follicle stem cells isolated from hair follicles. Totipotent stem cells have also been used to study whole tooth regeneration. Ning et al. cultured mouse ES cells in ameloblast-conditioned medium to obtain ameloblast-like cells, which initiated the induction of embryonic stem cells into ameloblasts [18]. Cai et al. combined human iPS-derived epithelium with mouse tooth germ mesenchyme and implanted it into the renal capsules of nude mice to obtain tooth-like structures [19]. Ochiai et al. obtained Pitx1-positive odontogenic epithelial cells by adding FGF8 and BMP4 to the medium sequentially [20]. Mouse embryonic stem cells differentiated into odontogenic epithelium with the characteristics of ameloblasts when the timing of BMP4 expression was accurately regulated [21]. However, relatively few studies have examined the DE differentiation of human embryonic stem (hES) cells and their odontogenic potential at present [22, 23]. As original pluripotent stem cells, hES cells are derived from the inner cell mass of human blastocysts and have become one of the latest hot spots in stem cell research.

**Fig. 1:**
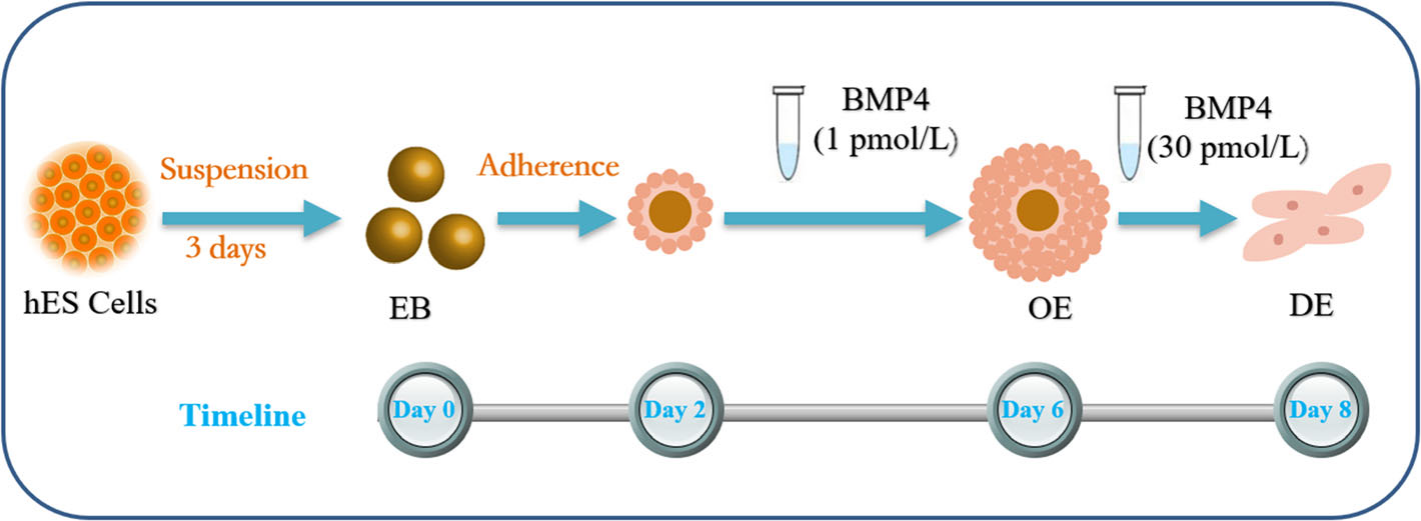
Human embryonic stem (hES) cell differentiation culture protocols. After hES cells were suspension-cultured for 3 days, embryoid bodies (EBs) formed (day 0). The EBs were transferred to standard culture plates and attached for 2 days (day 2). 1 pmol/L (pM) BMP4 stimulated EBs for 4 days to obtain oral ectoderm (OE) (day 6). Next, the OE differentiated into dental epithelium (DE) on stimulation with 30 pM BMP4 (Day 8)

In this study, hES cells were induced to differentiate into DE using sequential stimulation with different concentrations of BMP4 (Fig. 1). We then mixed the DE with ED14.5 mouse dental mesenchyme and transplanted it into the kidney capsules of nude mice. Several teeth with dentin and enamel formed after 30 days. This DE differentiation strategy may be important for tooth regeneration.

## Methods

### hES cell culture

hES cells were cultured in mTeSR1 (STEMCELL Technologies) containing supplement (STEMCELL Technologies) and 1% penicillin/streptomycin (Gibco) on six-well plates precoated with Matrigel (Corning). The culture plates were incubated at 37 °C (5% CO_2_, 95% air), and the medium was changed daily. The colonies were evaluated under an inverted microscope (Olympus).

### Differentiation of oral ectoderm (OE)

A low-attachment six-well plate (Corning) was used to form embryoid bodies (EBs) from hES cells. On reaching 60–70% confluence, the hES cells were dissociated with 1% dispase and cultured in EB medium [DME/F12 (HyClone) containing 20% KSR, 3% FBS, 1% MEM-NEAA (Gibco), 1% GlutaMAX (Gibco), 1% l-GlutaMAX (Gibco), 1% penicillin/streptomycin, and 0.0074% 2-mercaptoethanol (0.1 mmol/L)]. The culture plates were incubated at 37 °C (5% CO_2_, 95% air) for 3 days. The resulting EBs were transferred to standard six-well culture plates (1:1). After culture in EB medium for 2 days, 1 pmol/L (pM) BMP4 (Sigma-Aldrich) was added to the medium for 4 days. In the control group, no BMP4 was added. The culture medium was changed every other day, and EBs were evaluated under an inverted microscope.

### Differentiation of dental epithelium

After the hES cells had grown for 6 days by static adherence, the cells differentiated into OE. We increased the concentration of BMP4 and added 30 pM BMP4 to the medium for 2 days. In the control group, EB was cultured in the EB medium for 8 days. In another control group, the EB medium contained 1 pM BMP4 from day 2 to day 6, and no BMP4 from day 6 to day 8.

### Quantitative reverse-transcription polymerase chain reaction

Samples were subjected to quantitative reverse-transcription polymerase chain reaction (qRT-PCR) on an Mx3000P Real-Time PCR System (Agilent Technologies). The data were normalized to 28S rRNA expression. The primers used for qRT-PCR are presented in Additional file 1: Table S1.

### Immunofluorescence

Cells grown on glass culture plates were rinsed with phosphate-buffered saline (PBS), fixed with 4% paraformaldehyde for 30 min at room temperature (RT), and permeabilized with 0.1% (v/v) Triton X-100 (Solarbio) for 30 min at RT. Then, the cells were incubated in 3% bovine serum albumin at RT for 2 h to block nonspecific binding. The substrates were incubated with Pitx1 (mouse, Santa Cruz Biotechnology), Dlx2 (mouse, Santa Cruz Biotechnology), AMBN (rabbit, Abcam), β-catenin (rabbit, Abcam), and p-Smad1/5/8 (goat, Santa Cruz Biotechnology) primary antibodies at RT for 2 h. Then, the cells were washed twice with PBS and incubated with secondary antibodies for 1 h in the dark. The nuclei were stained with 4′,6-diamidino-2-phenylindole (DAPI, 1 μg/mL; Sigma-Aldrich) for 5 min at RT. The cells were visualized with a laser scanning confocal microscopy (Nikon Corporation).

#### In vivo transplantation assay

ED14.5 mouse embryos were obtained by dissection from pregnant KM mice, and the tooth embryos were isolated under a stereomicroscope. The tooth embryos were washed with PBS and digested for 10.5 min with 100 mg/mL dispase; then, dental mesenchyme was isolated under a stereomicroscope. Day 8 epithelial cell aggregates were harvested and mixed with E14.5 mouse dental mesenchyme (mDM). The mixed tissues (1 × 10^6^ dental epithelial cells + 4 mDM) were transplanted into the renal capsules of 6-week-old nude mice, which were raised for 30 days.

### Statistical analysis

The data are presented as the mean ± standard deviation (SD). Using Origin 2019 software, one-way analysis of variance (ANOVA) followed by Tukey’s test was performed to assess significant differences among groups. *P* values < 0.05 and < 0.01 indicated statistical significance and high statistical significance, respectively.

## Results

### A low BMP4 concentration promotes hES cell differentiation into OE

**Fig. 2:**
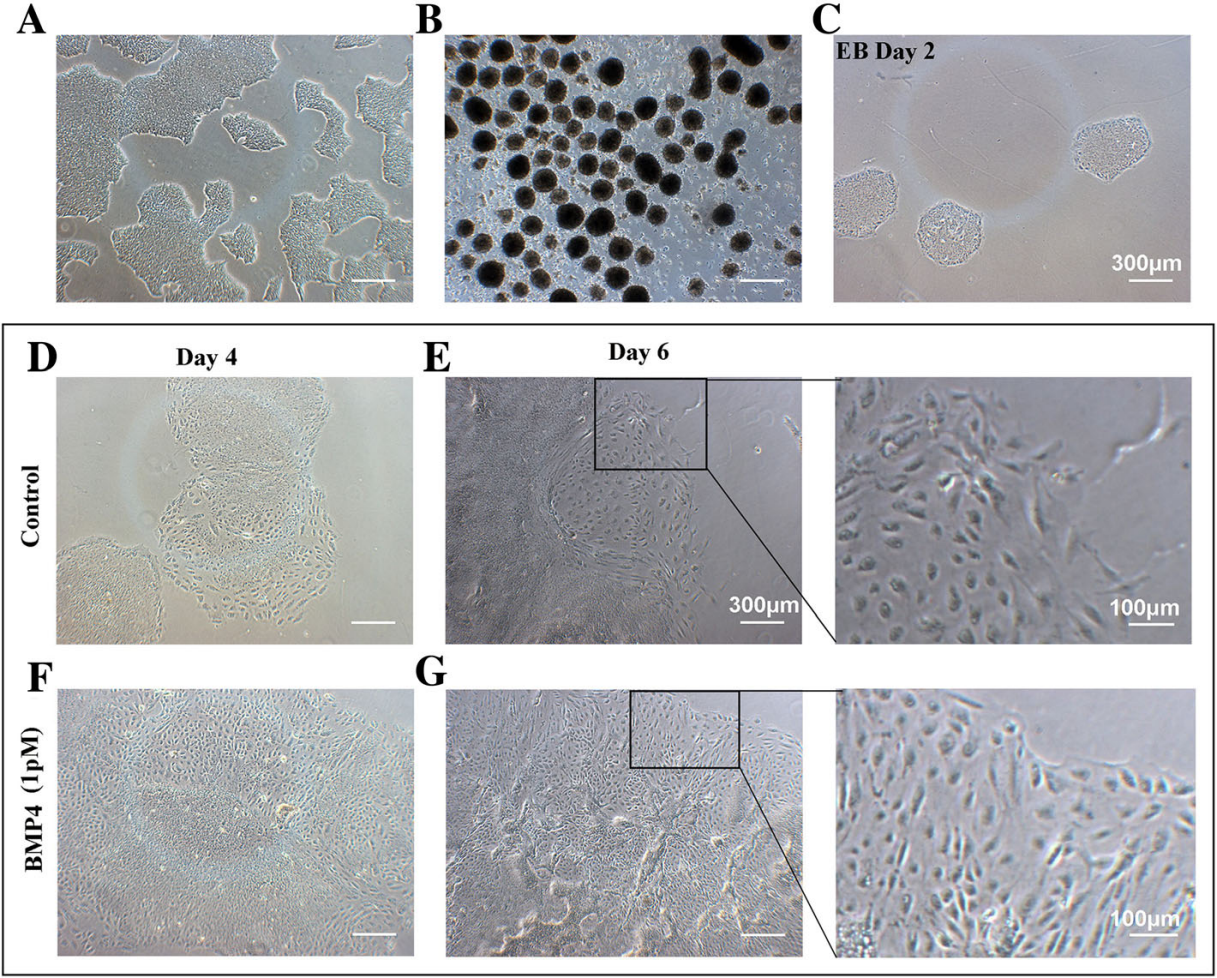
Cell morphology. **a** The cells were used to form EBs 3 days after passage. **b** hES cells formed EBs after suspension culture for 3 days. **c** The EBs were transferred to standard culture plate for 2 days and some EBs attached. The morphology of EBs attached for 4 (**d**) and 6 (**e**) days in EB medium. The cells were stimulated with 1 pM BMP4 in EB medium on days 4 (**f**) and 6 (**g**). Scale bar, 300 μm

Under the microscope, the edges of the hES cell colonies were clear and bright and slightly elevated. The cells were small and closely arranged, and there was no obvious boundary between the cells. On the third day, the cell confluence exceeded 60%, which is suitable for EB seeding (Fig. 2a).

The cells were suspended in low adhesion culture plates for 2 days, and embryoid bodies (EBs) formed. When suspended for 3 days (Fig. 2b), the EBs were transferred to standard culture plates. Two days later, most of the EBs were attached, although a few cells had migrated out of the EBs (Fig. 2c). On day 6, more cells had migrated and expanded (Fig. 2e, g). Compared with the control, more cells induced with 1 pM BMP4 migrated and these cells were slender and similar to epithelioid cells (Fig. 2g).

**Fig. 3:**
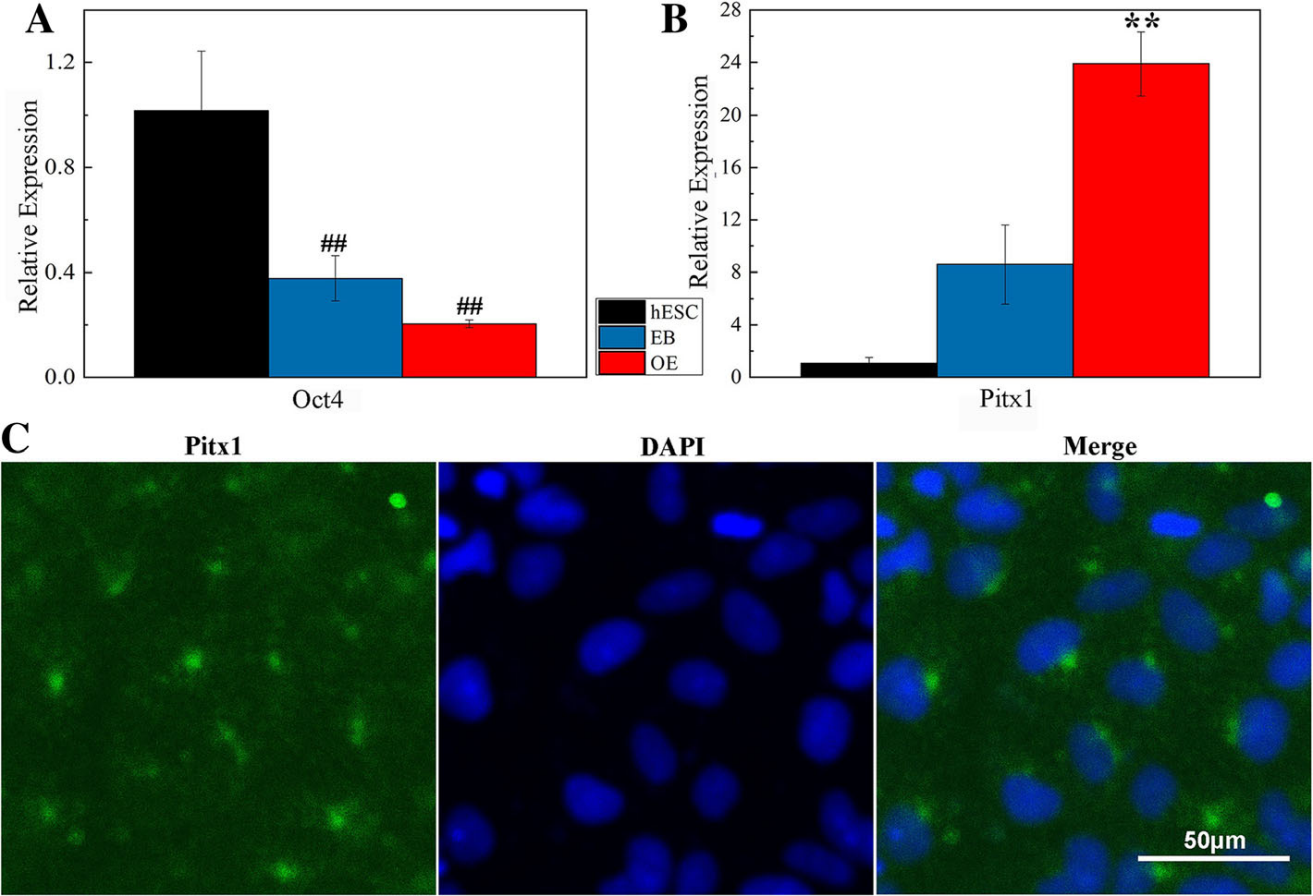
A low BMP4 concentration stimulated hES cells to differentiate into Pitx1+ cells. qRT-PCR expression levels of Oct4 (**a**) and Pitx1 (**b**) genes. EBs were cultured in EB medium for 6 days, and OE was cultured with 1 pM BMP4. ^##^*P* < 0.01 vs. hES cells; **P < 0.01 vs. EB. **c** Immunostaining of OE, DAPI (blue), and Pitx1 (green). Scale bar, 50 μm

The expression of the stem gene and OE-related genes was detected. On day 6, the expression of Oct4 was significantly reduced (Fig. 3a). The reduced level of stemness indicated that the cells had differentiated. Compared with EBs (control), the expression of Pitx1 increased 2.8 times with 1 pM BMP4 for 4 days (Fig. 3b). Pitx1 is a marker of OE [20]. Immunostaining indicated that Pitx1 was expressed in the hES cell-derived OE stimulated with 1 pM BMP4 (Fig. 3c). On induction with a low concentration of BMP4, hES cells differentiated into Pitx1-positive OE.

### High BMP4 concentration promotes OE differentiation into DE

**Fig. 4:**
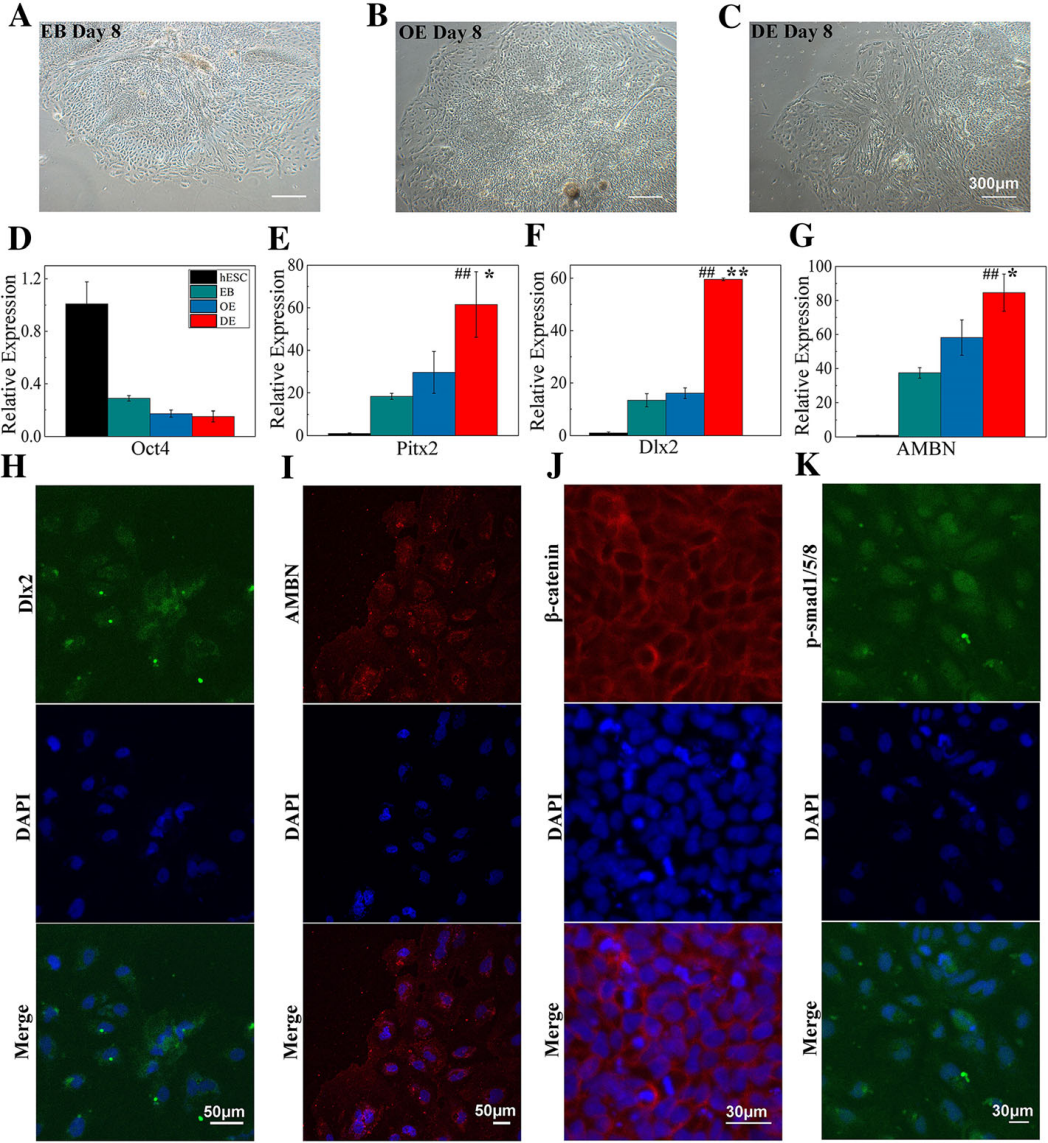
Dental epithelium-like cells were obtained with a high concentration of BMP4. **a** EB was cultured in EB medium for 8 days (day 8). **b** OE was cultured in EB medium for 2 days. **c** OE was stimulated with 30 μM BMP4 for 2 days. Scale bar, 300 μm. qRT-PCR expression levels of Oct4 (**d**), Pitx2 (**e**), Dlx2 (**f**), and AMBN (**g**). *P < 0.05 vs. OE; **P < 0.01 vs. OE; ^##^*P* < 0.01 vs. EB. **h**, **i** Immunostaining of day 8 cells stimulated with 30 μM BMP4: DAPI (blue), Dlx2 (green), and AMBN (red). Scale bar, 50 μm. The DE-like cells expressed β-catenin (**j**) and p-Smad1/5/8 (**k**). DAPI (blue), β-catenin (red), p-Smad1/5/8 (green). Scale bar, 30 μm

On day 8, the migration of spindle-shaped cells increased (Fig. 4a–c). The expression of Oct4 decreased significantly (Fig. 4d). qRT-PCR showed that the expression of Pitx2, Dlx2, and AMBN was 2.1, 3.7, and 2.7 times higher in the BMP4-treated DE than in the OE control group, respectively (Fig. 4e–g). An early marker of tooth development, Pitx2 expression is restricted to the developing DE and is specifically expressed in the dental lamina [24]. Dlx2 is a marker of DE, DM, and the hypothalamus [20]. AMBN, a marker of ameloblasts, is specifically expressed in the enamel matrix and ameloblasts [25]. The immunofluorescence results indicated that stimulation with a high concentration of BMP4 promoted the differentiation of Dlx2+/AMBN+ DE-like cells (Fig. 4h, i).

To investigate which signaling pathways are involved in the differentiation of DE, we examined the expression of key proteins in the Wnt/β-catenin and Bmp signaling pathways. On stimulation with different concentrations of BMP4, β-catenin and p-Smad1/5/8 were both expressed in the DE-like cells (Fig. 4j, k).

### Tooth-like structures generated from hES cell-derived DE

**Fig. 5:**
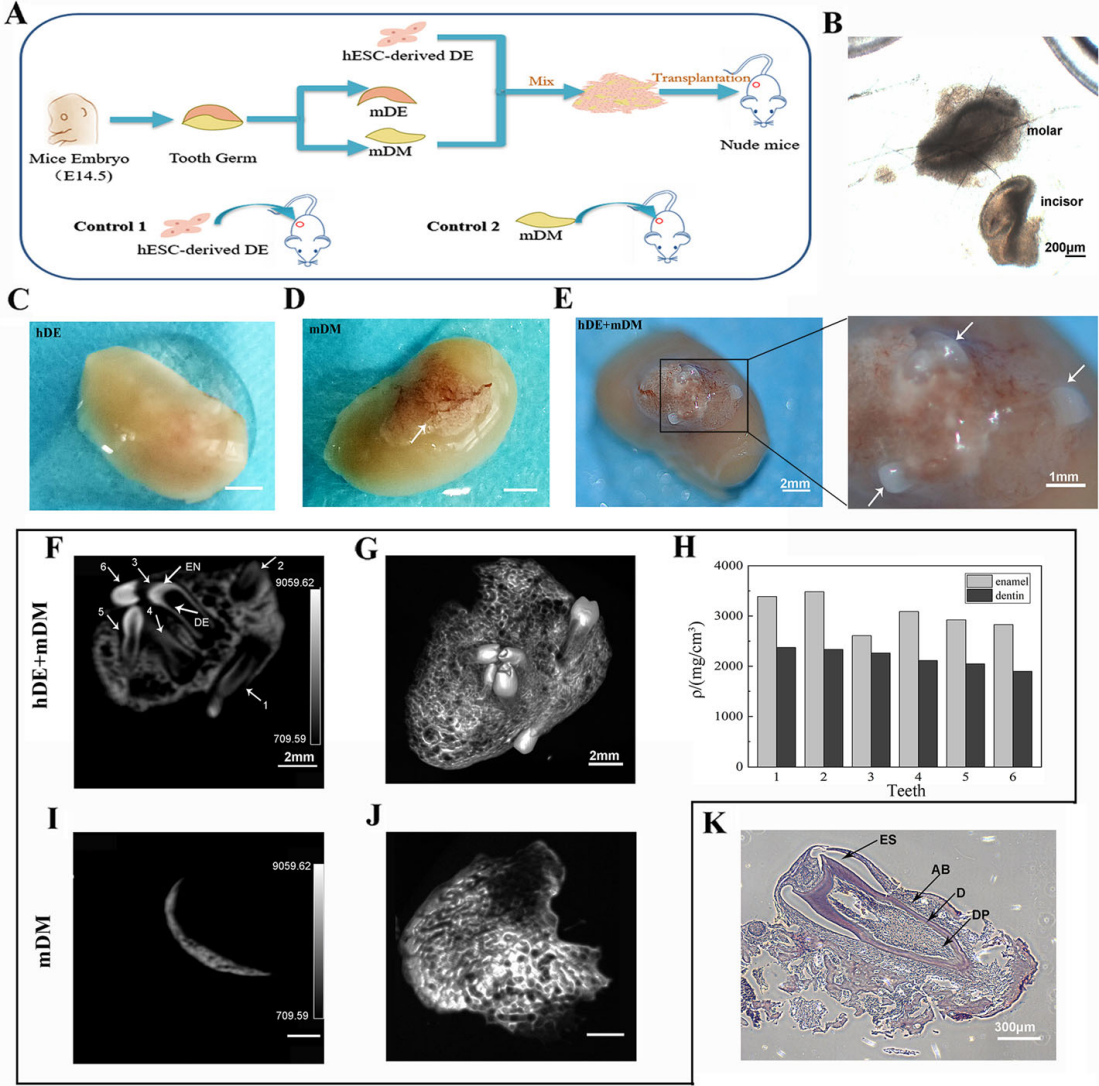
Tooth-like structures formed 30 days after introducing hDE-like cells mixed with mDM in vivo. **a** The in vivo experimental scheme. DE-like cells were transplanted into the renal capsules of nude mice after mixing with mDM. **b** The mDM isolated from embryos. Scale bar, 200 μm. Thirty days after transplantation, the kidneys were removed. Kidneys transplanted with DE-like cells (**c**), mDM (**d**), and DE-like cells + mDM (**e**). Scale bar, 2 mm. Micro-CT of kidneys with transplants of mDM (**i**) and DE-like cells + mDM (**f**). DE, dentin; EN, enamel. Scale bar, 2 mm. **j** Some cementoid formed on the kidneys in the mDM group. **g** 3D images of the six teeth that formed after DE-like cells and mDM were transplanted. Scale bar, 2 mm. **h** Intensity of the enamel and dentine in the six teeth. **k** Hematoxylin and eosin staining showed tooth-like structures containing alveolar bone (AB), enamel space (ES), dentin (D), and dental pulp (DP). Scale bar, 300 μm

Under a stereomicroscope, we isolated mouse molar germ and incisor germ (Fig. 5b). We detached mouse DE (mDE) from the dental germ and mixed the human DE (hDE) with mouse DM (mDM). hDE+mDM, hDE, and mDM were transplanted into the renal capsules of nude mice (Fig. 5a; Additional file 4: Figure S4). Thirty days after transplantation, tooth-like structures had formed on the kidneys of two of the nine mice transplanted with hDE+mDM (Fig. 5e). In addition, a white substance formed in the kidneys of all of the mDM mice (Fig. 5d). A white substance was also found in the livers of some hDE+mDM mice (Additional file 4: Figure S4). The kidneys of the hDE mice were very smooth, and nothing formed (Fig. 5c).

On micro-computed tomography (CT) examination, we found that teeth formed only in the hDE+mDM mice. On the kidneys of mDM mice, mineralization may have formed (Fig. 5i, j). Six teeth similar to incisors formed in the kidneys (Fig. 5f, g). The intensity of the enamel and dentine was calculated (Fig. 5h). The results indicate that hES cell-derived DE-like cells have the potential to form teeth with enamel and dentin. Moreover, hematoxylin and eosin staining revealed that the newly generated tooth-like structures contained dentin, dental pulp, enamel space, and alveolar bone, similar to natural teeth (Fig. 5k).

## Discussion

Tooth development is a process in which the epithelium and mesenchyme interact and induce each other [26]. A signal of odontogenic potentiality first appears in the future DE. As the tooth germ develops, the epithelium loses its odontogenic potential, while the underlying mesenchyme develops odontogenic potential, inducing tooth germ development [27].

We found that a low concentration of BMP4 stimulated hES cells to differentiate into oral ectoderm, and then, a high concentration of BMP4 caused the oral ectoderm to develop into DE [20]. hES cells were seeded into low-attachment six-well plates and formed EBs (1:1). When the EBs were cultured for 3 days, the expression levels of Pax6, Sox1, and FGF5 were the highest, while the expression of GATA 4 (a marker of endoderm) and T (a marker of mesoderm) was relatively low (Additional file 2: Figure S2). Pax6 and Sox1 are expressed in the ectoderm, whereas FGF5 is a gene of the preoral ectoderm. The EBs attached to standard culture plates, and most of the EBs remained attached on day 2. When the EBs were stimulated with 1 pM BMP4 for 4 days, Pitx1 expression increased significantly (Fig. 3; Additional file 3: Figure S3). As a result, we established a protocol for the induction of OE from hES cells. Then, the concentration of BMP4 was increased to 30 pM, and the OE differentiated into Pitx2+/Dlx2+/AMBN+ cells after culture for 2 days. On sequential stimulation with different concentrations of BMP4, DE-like cells were obtained from EBs in 8 days.

The classic Wnt/β-catenin signaling pathway plays an important role in tooth embryo development. When the Wnt signal is activated, β-catenin phosphorylation is inhibited, thus the unphosphorylated β-catenin cannot be hydrated and enter the nucleus to initiate transcription of related genes [28, 29]. BMP4 binds with the BMPRII receptor on the cell membrane and phosphorylates the BMPRI receptor. The phosphorylated BMPRI receptor forms a tetramer with two BMPRII receptors, which phosphorylates Smad1/5/8. The phosphorylated Smad1/5/8(p-Smad1/5/8) binds with Smad4 and is transferred into the nucleus to regulate gene expression [30–32]. Induced with different concentrations of BMP4, β-catenin and p-Smad1/5/8 were expressed and the cellular Wnt/β-catenin and Bmp signaling pathways were activated.

Finally, we confirmed the odontogenic potential of hES cell-derived DE in vivo. Thirty days after transplanting hDE+mDM, six white tooth-like structures were seen on the surfaces of kidneys. These teeth had enamel and dentin, and an incisor-like morphology. However, the rate of odontogenesis in vivo was relatively low (2 of 9 mice). Perhaps, too few Pitx2+/Dlx2+/AMBN+ cells were transplanted into the nude mice. Although different concentrations of BMP4 sequentially stimulated hES cells to differentiate into DE-like cells, only the peripheral EBs were Pitx2+/Dlx2+/AMBN+ cells.

In the future, we will combine BMP4 and other signaling molecules to enhance DE differentiation. We hope to confirm the odontogenic potential in orthotopic transplantation experiments, which is important for the clinical regeneration of human teeth.

## Conclusion

In summary, we established an optimized strategy to induce the differentiation of dental epithelium from hES cells in vitro. On treatment with a low BMP4 concentration, hES cells differentiated into Pitx1+ oral ectoderm. When these were stimulated with a high BMP4 concentration, these cells then differentiated into Dlx2+/AMBN+ dental epithelium. Mixed with mouse E14.5 mesenchyme, the epithelium generated tooth-like structures with enamel and dentin. Therefore, hES cell-derived dental epithelium has odontogenic potential. This is important for the further study of tooth regeneration.

## Additional files

Additional file 1: **Table S1.** Presenting the primer used for qRT-PCR. (XLS 27 kb)

Additional file 2: **Figure S2.** EBs were suspension-cultured for 1, 3, 5, or 7 days. qRT-PCR expression levels of GATA4 (A), T (B), Pax6 (C), Sox1 (D), and FGF5 (E). ## *P* < 0.01, # *P* < 0.05 vs. hES cells. (TIF 245 kb)

Additional file 3: **Figure S3.** qRT-PCR expression levels of Pitx1 in BMP4-treated EBs. (A) EBs were induced with different concentrations of BMP4 for 4 days. (B) EBs were induced with 1 pM BMP4 for different numbers of days. ## *P* < 0.01 vs. hES cells; ** *P* < 0.01 vs. control. (TIF 157 kb)

Additional file 4: **Figure S4.** The kidney of a nude mouse was exposed surgically (A, B). There was some osteoid tissue on the liver in the hDE+mDM group after 30 days (C, D). (TIF 1174 kb)

## Data Availability

The datasets used and/or analyzed during the current study are available from the corresponding author on reasonable request.

## Abbreviations

hES: Human embryonic stem
DE: Dental epithelium
BMP4: Bone morphogenetic protein 4
OE: Oral ectoderm
qRT-PCR: Quantitative reverse transcription-polymerase chain reaction
DM: Dental mesenchyme
3D: Three-dimensional
iPS: Induced pluripotent stem
EBs: Embryoid bodies
mDE: Mouse dental epithelium
mDM: Mouse dental mesenchyme
CT: Computed tomography

## References

[1] Ameet MM, Avneesh HT, Babita RP (2013). The relationship between periodontitis and systemic diseases – hype or hope?. J Clin Diagn Res.

[2] Little JW (2008). Periodontal disease and heart disease: are they related?. Gen Dent.

[3] Zembic A, Wismeijer D (2014). Patient-reported outcomes of maxillary implant-supported overdentures compared with conventional dentures. Clin Oral Implant Res.

[4] Preoteasa E, Imre M, Preoteasa CT (2014). A 3-year follow-up study of overdentures retained by mini-dental implants. Int J Oral Maxillofac Implants.

[5] Duailibi SE, Duailibi MT, Vacanti JP (2006). Prospects for tooth regeneration. Periodontol..

[6] Murray PE, Garcia-Godoy F (2004). Stem cell responses in tooth regeneration. Stem Cells Dev.

[7] Hu B, Liu Y, Wang SL (2005). Tooth tissue engineering: from cells to organ, an odyssey far from finished. Shanghai J Stomatol.

[8] Peterkova R, Hovorakova M, Peterka M (2014). Three-dimensional analysis of the early development of the dentition. Aust Dent J.

[9] Jia SH, Zhou J, Gao Y (2013). Roles of Bmp4 during tooth morphogenesis and sequential tooth formation. Development..

[10] Aberg T, Wozney J, Thesleff I (1997). Expression patterns of bone morphogenetic proteins (Bmps) in the developing mouse tooth suggest roles in morphogenesis and cell differentiation. Dev Dyn.

[11] Nakao K, Morita R, Saji Y (2007). The development of a bioengineered organ germ method. Nat Methods.

[12] Ono M, Oshima M, Ogawa M (2017). Practical whole-tooth restoration utilizing autologous bioengineered tooth germ transplantation in a postnatal canine model. Sci Rep.

[13] Zhang W, Ahluwalia IP, Yelick PC (2010). Three dimensional dental epithelial-mesenchymal constructs of predetermined size and shape for tooth regeneration. Biomaterials..

[14] Zhang W, Vazquez B, Yelick PC (2017). Bioengineered post-natal recombinant tooth bud models. J Tissue Eng Regen Med.

[15] Komine A, Suenaga M, Nakao K (2007). Tooth regeneration from newly established cell lines from a molar tooth germ epithelium. Biochem Biophys Res Commun.

[16] Anderson TR, Toverud SU, Yung RCW (1982). Separation and partial purification of acid phosphatases of the enamel organ of rat molars. Archives Oral Biology.

[17] Arakaki M, Ishikawa M, Nakamura T (2012). Role of epithelial-stem cell interactions during dental cell differentiation. J Biol Chem.

[18] Ning F, Guo Y, Tang J (2010). Differentiation of mouse embryonic stem cells into dental epithelial-like cells induced by ameloblasts serum-free conditioned medium. Biochem Biophys Res Commun.

[19] Cai J, Zhang Y, Liu P (2013). Generation of tooth-like structures from integration-free human urine induced pluripotent stem cells. Cell Regeneration.

[20] Ochiai H, Suga H, Yamada T (2015). BMP4 and FGF strongly induce differentiation of mouse ES cells into oral ectoderm. Stem Cell Res.

[21] Zhang Y, Li Y, Shi R (2017). Generation of tooth-periodontium complex structures using high-odontogenic potential dental epithelium derived from mouse embryonic stem cells. Stem Cell Res Ther.

[22] Bluteau G, Luder HU, De BC (2008). Stem cells for tooth engineering. Eur Cells Mater.

[23] Yen AHH, Sharpe PT (2008). Stem cells and tooth tissue engineering. Cell Tissue Res.

[24] Ge XL, Wang BM, Xu X (2012). Roles of Pitx2 during early development of tooth germ. Chinese Bull Life Sci.

[25] Kobayashi K, Yamakoshi Y, Hu JC (2007). Splicing determines the glycosylation state of ameloblastin. J Dent Res.

[26] Pispa J, Thesleff I (2003). Mechanisms of ectodermal organogenesis. Dev Biol.

[27] Yamamoto H (2003). Analysis of tooth formation by reaggregated dental mesenchyme from mouse embryo. J Electron Microsc.

[28] Jarvinen E, Salazarciudad I, Birchmeier W (2006). Continuous tooth generation in mouse is induced by activated epithelial Wnt/beta-catenin signaling. Proc Natl Acad Sci USA.

[29] Jager M, Dayraud C, Mialot A (2013). Evidence for involvement of Wnt signalling in body polarities, cell proliferation, and the neuro-sensory system in an adult ctenophore. PLoS One.

[30] Nishimura R, Hata K, Ikeda F (2003). The role of Smads in BMP signaling. Front Biosci.

[31] Chen D, Ji X, Harris MA (1998). Differential roles for bone morphogenetic protein (BMP) receptor type IB and IA in differentiation and specification of mesenchymal precursor cells to osteoblast and adipocyte lineages. J Cell Biol.

[32] Shi Y, Massague J (2003). Mechanisms of TGF-beta signaling from cell membrane to the nucleus. Cell..

