# Supplementary figures and images for: Sequential stimulation with different concentrations of BMP4 promotes the differentiation of human embryonic stem cells into dental epithelium with potential for tooth formation
Source: Stem Cell Res Ther. 2019 Aug 29;10:276. doi: 10.1186/s13287-019-1378-7 (PMC6714076; doi:10.1186/s13287-019-1378-7)

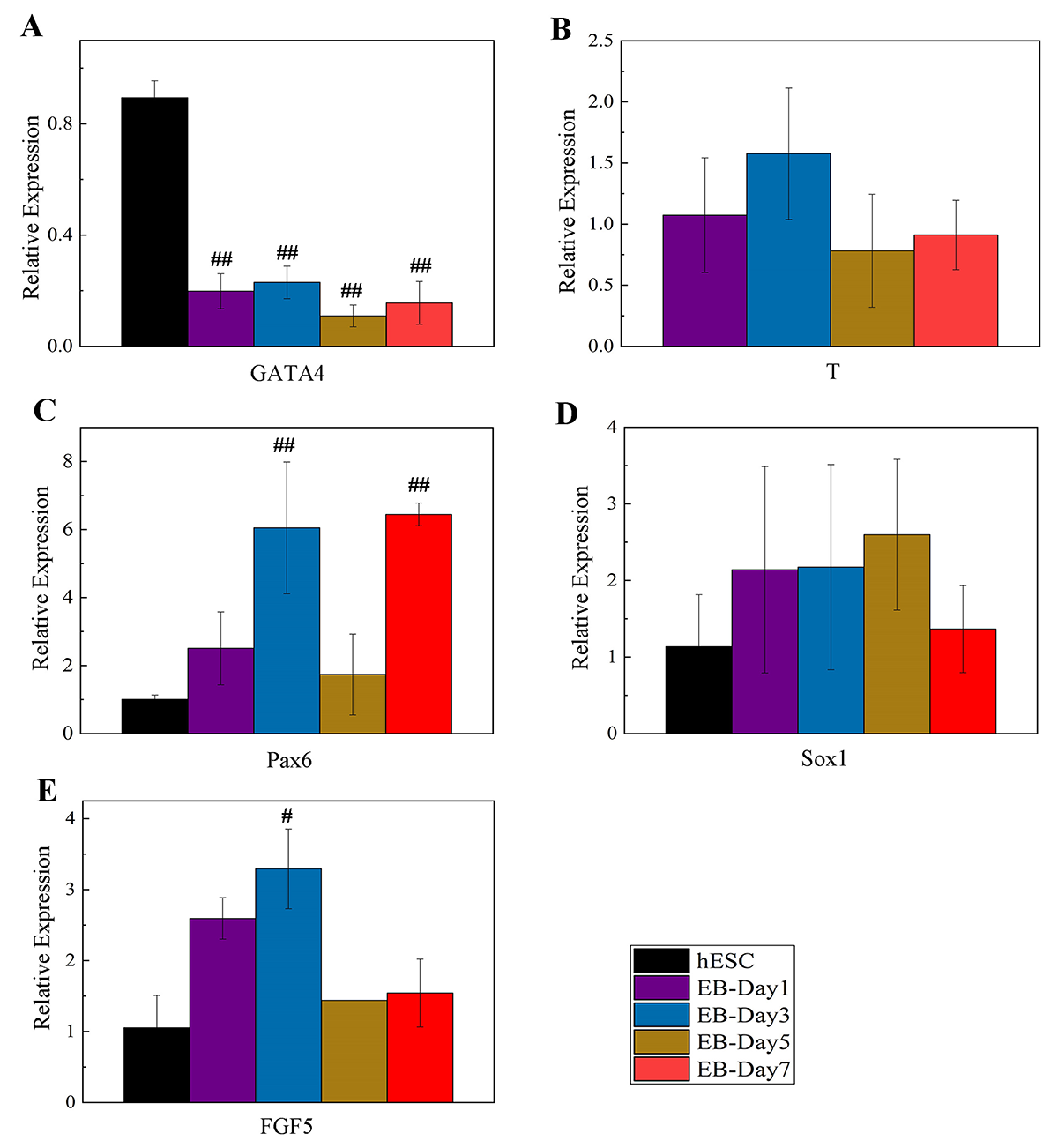

Supplement: Supplementary file 2 — Figure S2. EBs were suspension-cultured for 1, 3, 5, or 7 days. qRT-PCR expression levels of GATA4 (A), T (B), Pax6 (C), Sox1 (D), and FGF5 (E). ## P < 0.01, # P < 0.05 vs. hES cells. (TIF 245 kb) [file 13287_2019_1378_MOESM2_ESM.tif]

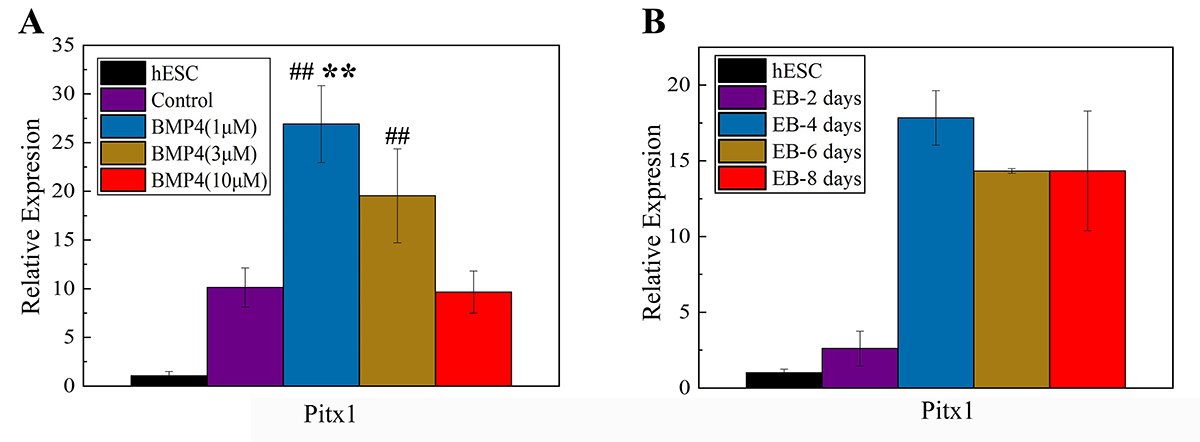

Supplement: Supplementary file 3 — Figure S3. qRT-PCR expression levels of Pitx1 in BMP4-treated EBs. (A) EBs were induced with different concentrations of BMP4 for 4 days. (B) EBs were induced with 1 pM BMP4 for different numbers of days. ## P < 0.01 vs. hES cells; ** P < 0.01 vs. control. (TIF 157 kb) [file 13287_2019_1378_MOESM3_ESM.tif]

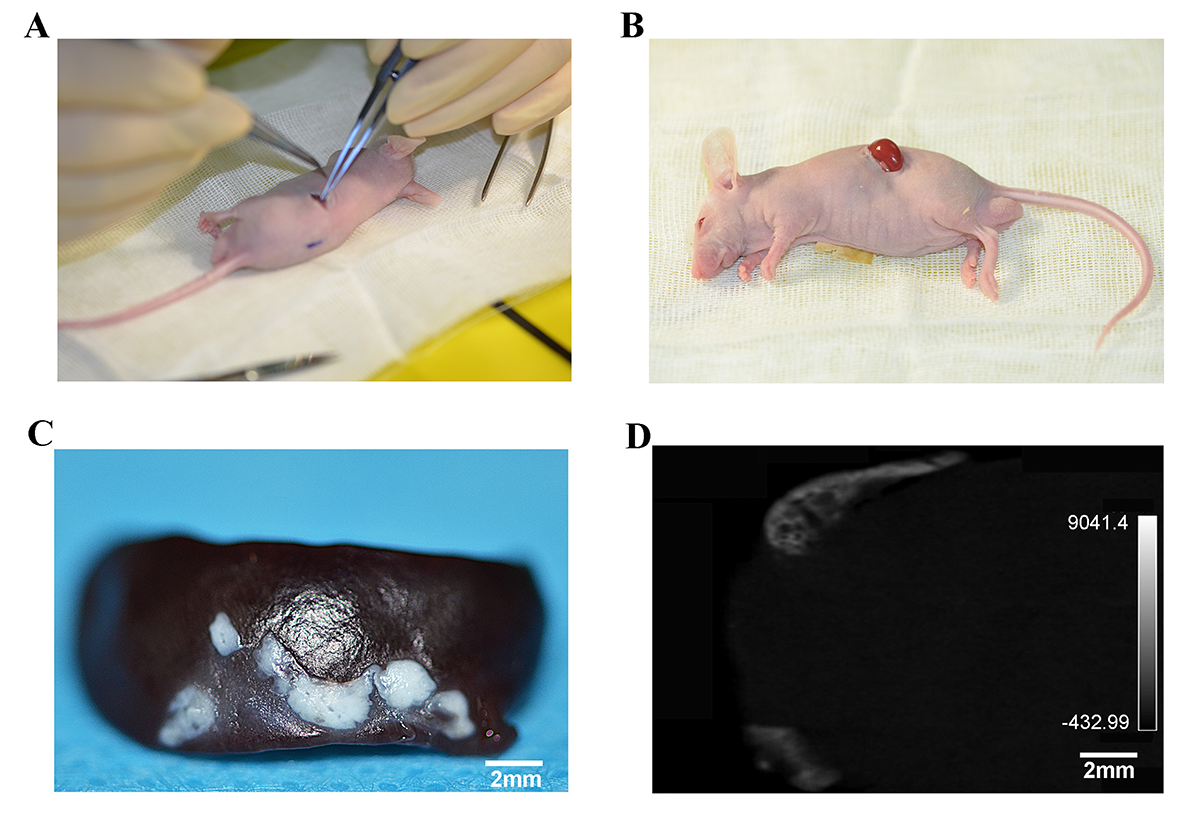

Supplement: Supplementary file 4 — Figure S4. The kidney of a nude mouse was exposed surgically (A, B). There was some osteoid tissue on the liver in the hDE+mDM group after 30 days (C, D). (TIF 1174 kb) [file 13287_2019_1378_MOESM4_ESM.tif]
